# Influencing Mechanism of Titanium-Extracted Tailing Slag on the Strength of CaO Steel Slag Hardened Paste

**DOI:** 10.3390/ma16030937

**Published:** 2023-01-19

**Authors:** Song Tang, Tongjiang Peng, Hongjuan Sun, Wenjin Ding, Liming Luo

**Affiliations:** 1 School of Environment and Resources, Southwest University of Science and Technology, Mianyang 621010, China; 2 Institute of Mineral Materials and Application, Southwest University of Science and Technology, Mianyang 621010, China; 3 Key Laboratory of Solid Waste Treatment and Resource Recycle, Ministry of Education, Mianyang 621010, China

**Keywords:** CaO, titanium-extracted tailing slag, steel slag, hardened paste, unconfined compressive strength

## Abstract

Hardened pastes with different mass percentages of steel slag (SS)/titanium-extracted tailing slag (TETS) were prepared under fixed CaO content to determine the influencing mechanism of TETS on the strength of CaO SS hardened paste. Furthermore, the effects and laws of curing time and SS/TETS ratios on the strength of hardened pastes were also investigated in this study. Importantly, hydration products, microstructures and the micro-area compositions of hardened pastes were analysed using X-ray diffraction, Fourier-transform infrared spectroscopy and scanning electron microscopy–energy dispersive spectrometer, respectively, to reveal the influencing mechanism of TETS on the CaO SS hardened pastes. The results demonstrated that the early strength of hardened pastes increases considerably following the inclusion of TETS. Specifically, the strength of the sample with an SS/TETS ratio of 22.5:67.5 at 1 d can be increased by more than 14 times. Notably, its strength at 90 days reached 19.36 MPa. Moreover, the diffraction peaks of calcite and C-S-H in the samples were also strengthened. Meanwhile, a diffraction peak of hydrocalumite appeared, and the calcites in the samples were curled up. When the SS/TETS ratio was equal to or more than 45:45, a diffraction peak of Ca(OH)_2_ appeared in the sample. Only a diffraction peak of Ca(OH)_2_ and weak diffraction peaks of calcite and C-S-H were observed in the samples without TETS, but there was no diffraction peak of hydrocalumite. The strength at 90 days was only 4.92 MPa. The increased strength of the hardened paste is closely related to the production of new phases after adding TETS. Solid particles in the hardened paste are cemented into a whole because of the hydration of C-S-H. Calcite forms the skeleton of the hardened pastes, whereas hydrocalumite fills in the pores among particles in hardened pastes, thus making them more compacted. As a result, there is increased.

## 1. Introduction

China is a global leader in infrastructure and steel production, with the stacking volume of steel slag (SS) exceeding 1 billion tons [1,2] and an annual growth amount of about 100 million tons [3,4]. However, the utilisation rate remains only about 30% [2,4]. The stacking of SSs occupies a large land area, causes environmental pollution [5,6], and burdens steel enterprises economically. Therefore, increasing the recycling use of SSs is critical to ensure the cyclic development of the steel industry.

As a primary by-product of the steel industry, SS primarily comprises C_2_S, C_3_S and C_4_AF [4,7]. The mineral composition of SS is identical to that of the cement composition [8,9,10]. However, the direct use of SS is problematic in building material production due to the low activity [11,12,13,14]. SS is mainly used as an auxiliary binding material [15,16]. However, auxiliary binding materials still need cement as a binder. Cement production is an energy-consuming and high-pollution process. It squanders excessive energy and natural resources and may discharge numerous greenhouse gases, thus contributing to the global greenhouse effect [17]. In particular, replacing cement with alkali-activated cementing materials has become a low carbon, energy-saving and environmentally friendly option in the background of global “carbon peak” and “carbon neutralisation”.

Some studies have highlighted that SS could be a precursor to alkali-activated cementing materials [18,19,20]. SS is activated by sodium silicate, and sodium silicate modules significantly impact hydration products and the strength of hardened pastes. The main hydration products of hardened pastes are C-S-H and Ca(OH)_2_ [21]. The nano-SiO_2_ can enhance the early strength of the SS mineral slag base activated by sodium silicate. The central enhancing mechanism is that nano-SiO_2_ can increase the compaction of the base by using its filling and crystal nucleus effects and producing fibroid C-S-H surrounding nanoparticles, thus increasing the strength of samples [22]. Cement-free SS binding materials were prepared using SS, complexing agent, aggregates and additives. Consequently, the compressive strength can reach 70 MPa. The primary strength formation mechanism is the development of pilo-taxitic needle-like sulfo-aluminates and villiform flocculation calcium silicate sol around the SS [23]. Sodium carbonate was used to activate the mixture of mineral slag and SS. Besides the C-(N)-S-A-H phase, hydrotalcite and calcite are produced in alkali-activated cementing materials. When the sodium carbonate content is 5%, the strength at 28 d is the highest, approaching 40 MPa [24]. One or a combination of several activators of 4% sodium sulphate, 1% sodium hydroxide and 1% sodium silicate was added to the SS concrete. It was revealed that the strength of SS concrete at 28 d can be increased by more than 23% upon the simultaneous addition of all three activators [25].

Although alkali-activated SS by sodium hydroxide and sodium silicate can develop relatively high compressive strength, it poses safety risks to operators and exhibits poor environmental characteristics because of the strong alkalinity and corrosion [26,27]. The use of activators such as sodium carbonate or sodium sulphate has many disadvantages, such as high cost, low strength and slow growth of strength. As a binding material used earlier, the benefits of CaO include extensive sources, environmental friendliness and low price. CaO-related research and applications continue to attract significant attention [17,28]. Although the micro-powder was prepared by SS through mechanical activation, its hydration reactivity can also be enhanced to a certain extent [9,29]. However, mechanical activation drives up material costs. Furthermore, hardened pastes prepared by CaO-activated SS powder following mechanical activation also present some challenges, such as low early strength, slow growth of strength and poor practicability, among others. Although adding mineral slag and other high-active components can increase the strength of hardened pastes, the high-activity mineral slag powder is costly in China at nearly 200 dollars/ton.

Titanium-extracted tailing slag (TETS) is the chlorinated tailing slag obtained from titanium extraction performed by high-temperature carbonation–low temperature chlorination. Its major composition is like that of mineral slag. The content and chemical reactivity of silica-aluminium vitreous body are relatively high, but the material cost is meagre, almost zero. Because chloridion in water exceeds the requirements of cement and concrete on the chlorine content in mixing materials, it is difficult to use in cement and cement concrete. At present, SS is mainly stacked, and it is urgent to establish a recycling use method.

To boost the early strength of CaO-SS binding materials, the low-cost and high-activity TETS is mixed into the CaO-SS based on the idea of solid waste synergism under the premise of no changes to the type and contents of the activator and no increase in material costs. The CaO SS/TETS composite cementing material was prepared on this foundation. It increases the activity of cementing materials and lowers material expenses. The strength of hardened pastes of CaO-SS based cementing material is improved at a low price to fulfil the requirements of pavement sub-base or base materials. Moreover, the influencing mechanism of TETS on the strength of the CaO-SS hardened pastes was determined. Research conclusions provided theoretical and experimental references for the recycling use of both TETS and SS.

## 2. Experiments

### 2.1. Materials

The list of experimental materials used in this study is as follows. TETS was collected from Panzhihua Iron & Steel Research Institute Co., Ltd. (Panzhihua, China). SS, aged for half a year, was obtained from Changgang Steel Plant (Jiangyou, China). It was then ground into powder to 200 meshes. The analytically pure CaO produced by Sichuan Chengdu Colon Chemical Co., Ltd. (Chengdu, China) was used in this study. Drinking tap water was used as experimental water.

**Figure 1:**
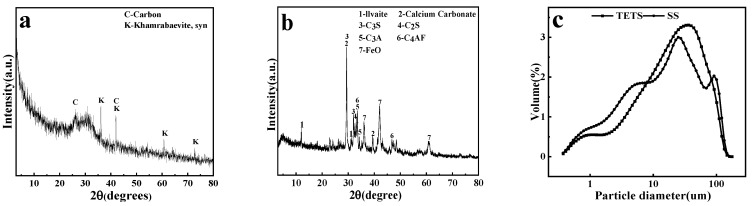
XRD patterns of TETS (**a**) and SS (**b**), particle size of TETS and SS (**c**).

Table 1 displays the chemical composition analysis results of raw materials. Figure 1a,b illustrate the X-ray diffraction (XRD) spectra of all raw materials. Figure 1a shows that an amorphous phase dominates TETS and the main crystal phases include titanium carbide (TiC) (d_002_ = 2.1564 Å) and graphitic-like carbon (d_002_ = 3.3970 Å). SS has relatively complicated crystal phases, primarily including ilvaite (d_110_ = 7.3145 Å), dicalcium silicate and tricalcium silicate (d_104_ = 3.0401 Å), brownmillerite (d_141_ = 2.6552 Å), ferric oxide (d_200_ = 2.1545 Å), and so on. Figure 1c shows the particle size test results of TETS and SS. For TETS, d_50_ = 21.76 μm and d_50_ = 17.50 μm for SS.

### 2.2. Experimental Programmes

The CaO mass percentage was fixed at 10% and the SS/TETS mass percentages were 90:0, 67.5:22.5, 45:45, 22.5:67.5 and 0:90 (the samples were numbered as S90T0, S67.5T22.5, S45T45, S22.5T67.5 and S0T90, respectively). Table 2 presents the dry material composition of the samples. The corresponding raw materials were weighed accurately and then poured into a cement mortar mixer, stirring slowly for 5 min and then quickly for 10 min. The above stirring process was repeated by the same method and for a specific period. The evenly stirred mixing materials were sealed up in a plastic bag for digestion for 5 h, followed by a standard compaction experiment. In this way, the optimal moisture content and maximum dry density of samples were determined. Under these parameters, the samples were moulded and cured to test their strength. All experiments were performed according to the Chinese Ministry of Transport standard (JTG E51-2009). The hardened pastes used a cylinder with H = D = 50 mm. Six specimens were prepared in each group. The average compressive strength and the absolute value of the difference between the average compressive strength and the maximum and minimum specimens were taken as positive and negative error values, respectively, to make the strength curves and error lines of different proportions of SS/TETS and different curing ages. Samples with strength close to the average value were frozen and dried for 72 h. Appropriately sized pieces were taken for the scanning electron microscopy–energy dispersive spectrometer (SEM–EDS) test. Additionally, the appropriate quantity of samples was taken and ground into powder for XRD and Fourier-transform infrared spectroscopy (FTIR) tests.

### 2.3. Sample Characterisation

The particle size distribution curve of TETS and SS powder was tested by Mastersize2000 laser particle size distribution meter. The particle size range was 0.02–2000 μm and the sensitivity was 0.2%. The Ultra X-ray diffractometer (D/MAX-IIIB, Rigaku, Japan) was used to test the phase compositions of all the used raw materials and hardened pastes under the following conditions: the Cu target, tube voltage = 40 kV, tube current = 40 mA, power = 2.2 kW, slit system = DS 1/2°, SS = 0.04 rad, AAS = 5.5 mm and scanning range = 3–80°. The INVENIO S-type infrared spectrometer was used for infrared spectroscopy. The wavenumber range was 3000–500 cm^−1^. The resolution, wavenumber accuracy and signal-to-noise ratio (SNR) were greater than 0.4 cm^−1^, 0.0005 cm^−1^ and 60,000:1, respectively. The Sigma300 field emission scanning electron microscope (ZEISS, Jena, Germany) was used to analyse the microstructures and components of solids. The MQS-2 pavement material strength tester was used to evaluate the strength of the hardened paste. The loading range, displacement measurement range, loading rate and measuring accuracy are 0–50 kN, 0–30 mm, 1 mm/min and ±1%, respectively.

## 3. Results and Discussion

### 3.1. Unconfined Compressive Strength of Hardened Pastes

**Figure 2:**
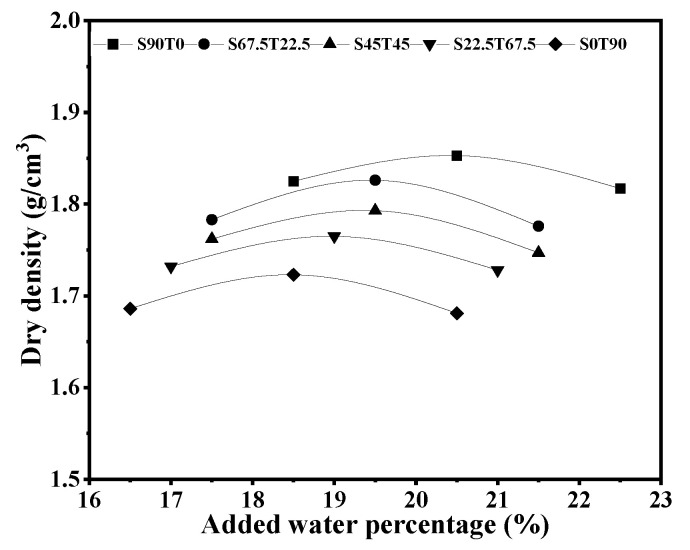
Experimental results of different proportions of standard compaction.

Figure 2 demonstrates the standard compaction experimental results under different SS/TETS ratios. It shows that both the optimal water-adding volume (moisture content) and the maximum dry density increase with increased SS content. The reasons are explained as follows. Because the SS powder has small particles and a large specific surface area, and its density is higher than that of TETS, the optimal moisture content increases slightly, along with the increase in the SS content, with the gradual increase in the maximum dry density.

**Figure 3:**
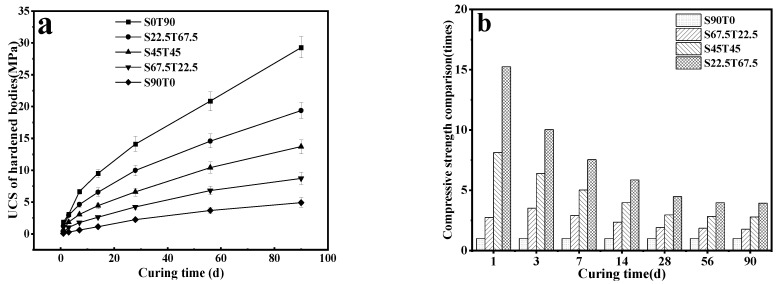
UCS of hardened body samples with different ratios at different ages (**a**) and strength multiplier with different ratios at different ages (**b**).

Figure 3 shows the strength (values) of hardened pastes with different SS/TETS ratios and growth rates of strength. Figure 3a specifically depicts the unconfined compressive strength (UCS) of hardened pastes. Figure 3b presents the strength ratios between other samples at different ages and S90T0 of the same age when the strength of S90T0 at different ages is set to 1. Figure 3a illustrates that the strength of hardened pastes is improved significantly after adding TETS. In particular, 1 day hardened pastes’ strength increases the most. The strong growth is positively related to TETS content and curing age. If hardened pastes have the same age, the strength increases with the increase of TETS contents: S0T90 > S22.5T67.5 > S45T45 > S67.5T22.5 > S90T0. The UCS of S22.5T67.5 and S45T45 at 7 days were 4.6 MPa and 3.1 MPa, respectively. These values are far greater than the 7-d strength of lime-fly ash stabilized materials and basically equal to the 7-d strength of cement-fly ash stabilized materials. However, the material costs of S22.5T67.5 and S45T45 are significantly lower.

Figure 3a shows that the growth rate of the strength of hardened pastes with different SS/TETS ratios is the highest within 7 d. The growth rate of strength is positively related to the TETS content. After 28 d, the growth rate of strength declines gradually. Such a reduction trend accelerates with the decrease in the TETS content. Compared with other samples, S0T90 still maintains a high strength growth rate after 28 d. Its UCS is the highest (S22.5T67.5) at the same age. Among all the samples, S90T0 exhibits the lowest growth rate of strength, and its UCS is the lowest at the same age. This outcome is mainly attributed to the low activity of SS. Because the hydration reactivity of TETS is far greater than that of the SS powder, the overall hydration reactivity of hardened pastes is enhanced after adding TETS and increases its strength accordingly.

Figure 3b shows that the strength of hardened pastes with different SS/TETS ratios and at different ages is increased following the inclusion of TETS. The strength rises more by adding more TETS, especially the early strength. The strengths of S22.5T67.5 at 1 d, 7 d, 28 d and 90 d are increased by 15.25, 6.54, 3.48 and 2.93 times compared to those of S90T0, respectively.

Table 3 presents the requirements of *Base Construction Technical Regulations for Highway Pavement* (JTGT F20-2015) on the minimum compressive strength standard (MCSS) and different mixing ratios of cement stable materials at 7 d.

Table 3 shows that, compared to the strength criteria of stable cement materials used most extensively in pavement base and sub-base in China, the strength of S45T45 can meet the strength requirements of pavement sub-base for all highway levels. S22.5T67.5 has a broader application range. However, the strength of the hardened paste without TETS (S90T0) cannot meet the requirements of the structural layer for any highway level. After adding TETS, the application range of the CaO SS powder can be expanded significantly. This activity can facilitate the recycling use of TETS and SS.

### 3.2. Phase Change of Hardened Pastes

**Figure 4:**
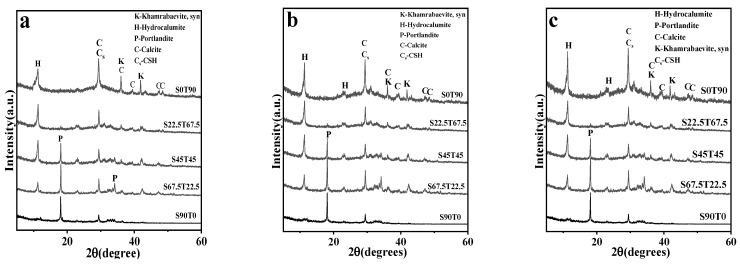
The XRD patterns of the hardened pastes with different ratios at 7 d (**a**), 28 d (**b**) and 90 d (**c**).

Figure 4 illustrates the XRD spectra of hardened pastes with different SS/TETS ratios at 7 d (Figure 4a), 28 d (Figure 4b) and 90 d (Figure 4c). Figure 4 shows that all hardened pastes except S90T0 have a characteristic peak of hydrocalumite (Cl-Ht) at ~2θ = 11.2°. This is a kind of hydration product of the silica–aluminium cementing material that contains chloride ions [30,31,32,33] and is also the main phase of chemical curing chloride ions. The XRD spectra of hydrocalumite differ significantly with the changes in the SS/TETS ratios. Among the hardened pastes of the same age, the diffraction peak of hydrocalumite is stronger with the increase in the TETS content. However, the diffraction peak of hydrocalumite is not observed in hardened pastes without TETS at different ages.

All hardened pastes with different SS/TETS ratios at various ages have strong diffraction peaks at ~2θ = 29.4°, indicating that their strength increases with the increase in the TETS content and curing age. For alkali-activated cementing materials, the diffraction peaks at ~2θ = 29.4° are superposing the diffraction peaks of calcite (crystal face 104) and C-S-H (or Cs-H) [17,34,35]. They are typical phases in hardened pastes of silica materials and are an essential component of the strength of hardened pastes [36]. C-S-H can bond loose solid particles in hardened pastes for a while and is the primary source of the early strength of hardened pastes. Small calcite particles can facilitate the growth of C-S-H, and large-sized calcites create the skeleton of hardened pastes. Together, they enable the increase in the strength of hardened pastes [37,38].

Besides, hardened pastes with a high SS content develop diffraction peaks of Portlandite at ~2θ = 18.1°; this diffraction peak intensity increases with the increase in the SS powder content. On the XRD spectra of S90T0 at different ages, the diffraction peak of calcium hydroxide is observed, but no diffraction peak of hydrocalumite is created. This outcome reveals that there is no diffraction peak of hydrocalumite in hardened pastes without TETS because of the low chlorine content. Furthermore, diffraction peaks of calcite and C-S-H are relatively faint, thus resulting in the lowest strength of S90T0 among all hardened pastes at the same age.

Based on the above experimental results, hardened pastes with different SS/TETS ratios develop four hydration reactions: (1) CaO first reacts with water to produce calcium hydroxide (Equation (1)), releasing an abundant amount of heat energy. (2) OH^−^, which is separated from calcium hydroxide through ionisation, gets distributed in the pore solution of hardened pastes. OH^−^ may also enter the vitreous body while corroding it on the base surface, thus disintegrating the vitreous body into a metastable state. Some calcium hydroxides react with active SiO_2_ in solid particles to produce hydration C-S-H gels (Equation (2)). (3) Some calcium hydroxides react with active Al_2_O_3_ and calcium chlorides in solid particles to produce hydrocalumite with chloride ion-curing function (Equation (3)). (4) Some other calcium hydroxides absorb CO_2_ in the air during curing to produce calcite particles (Equation (4)). These calcite particles develop the crystal nuclei of C-S-H and facilitate the crystallization of C-S-H gel [36,37,39]. The created C-S-H bonds hardened paste into an integral along with other gels. Some calcite crystals grow continuously to develop the base skeleton, thus improving the integrality and compressive strength of hardened pastes. After the reduction in the TETS content, there are insufficient vitreous bodies in hardened pastes to react with calcium hydroxides, thus resulting in residual calcium hydroxides. Furthermore, C_2_S and C_3_S in SS may produce some calcium hydroxides after hydration, thus increasing calcium hydroxide content in the hardened pastes. The main reaction equations are presented in Equations (1)–(4).

CaO + H_2_O = Ca(OH)_2_ (1)

Ca(OH)_2_ + SiO_2_ = CaSiO_3_↓ + H_2_O (2)

3Ca(OH)_2_ + Al_2_O_3_ + CaCl_2_ + 7H_2_O = 2Ca_2_ Al(OH)_6_ Cl(H_2_O)_2_↓ (3)

Ca(OH)_2_ + CO_2_ = CaCO_3_↓ + H_2_O (4)

### 3.3. FTIR Characteristics of Hardened Pastes

**Figure 5:**
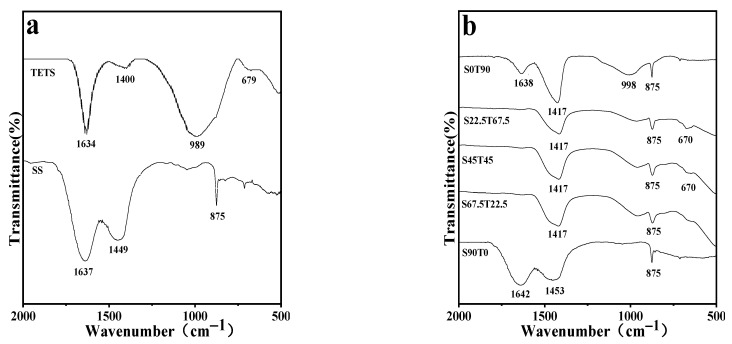

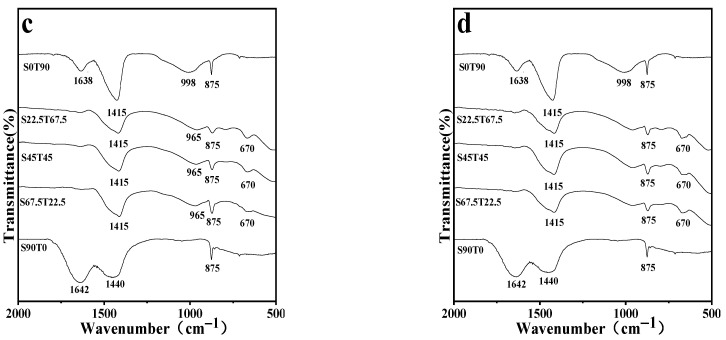
The FTIR spectra of TETS, SS (**a**), and hardened pastes at 7 d (**b**), 28 d (**c**), and 90 d (**d**) ages.

Figure 5 shows the FTIR spectra of hardened pastes with different SS/TETS ratios and at different ages. Only information within the wavenumber range of 2000–500 cm^−1^ is provided in the spectra to highlight effective data. Table 4 summarises the XRD spectra, relevant references, affiliations of absorption peaks and raw material components.

The absorption peak of S67.5T22.5 at the wavenumber of ~670 cm^−1^ is hardly developed at 7 d, but it begins to develop at 28 d, and a faint but noticeable absorption peak is developed at 90 d. This result indicates that the calcite content in samples increases with the curing time. The FTIR spectra of S90T0 at different ages change slightly, indicating that the structure of this hardened paste is relatively stable and exhibits no apparent changes in the curing period. This is because SS has low activity, resulting in the slow hydration reaction in hardened pastes. The quantity of calcite and C-S-H produced in the hardened paste is low, which is manifested in strength. Hence, the strength of S90T0 is lower than other hardened pastes at the same age.

Compared with other samples, the absorption peak intensity of S0T90 at the wavenumber of ~1638 cm^−1^ increases with curing age. This law is consistent with the variation laws of the diffraction peak of hydrocalumite in the XRD spectra. It reflects that the hydrocalumite content in samples increases with the increase in the curing time; increasing the curing time is beneficial to facilitate the production of hydrocalumite.

### 3.4. Micromorphology of Hardened Pastes

Table 3 shows that, when TETS replaces 50% of SS, hardened pastes strength can meet the highway base construction requirements for all levels. Besides, hardened pastes with different SS/TETS ratios have the same phase types. Hence, S45T45 was selected to test micromorphology at different ages and some microcell element composition was tested by EDS.

**Figure 6:**
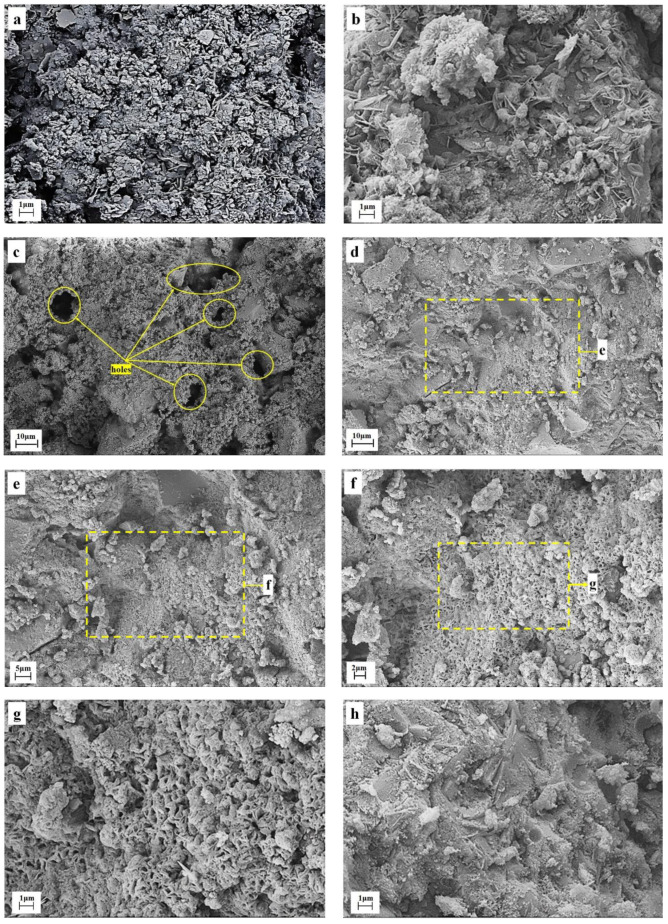

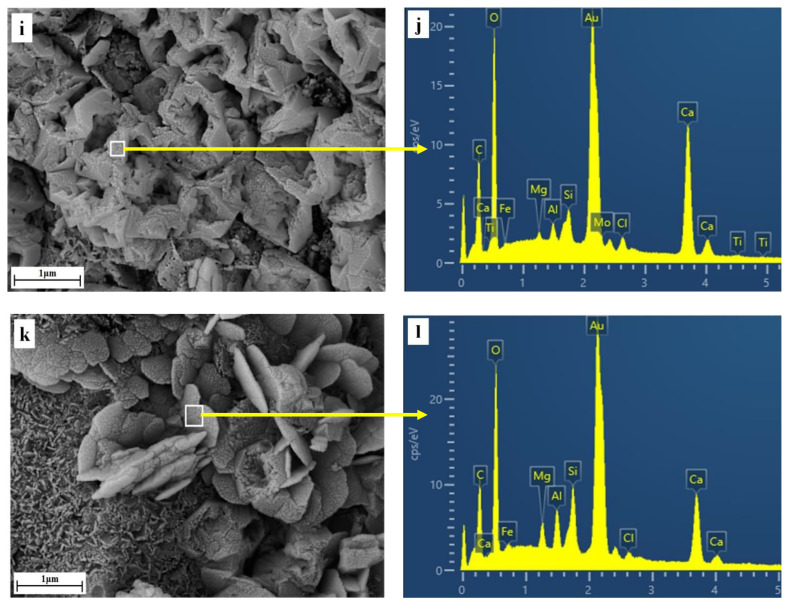
SEM and EDS of partial hardened samples. (**a**） shows the SEM images of S45T45 hardened samples at 7 days, (**b**) shows the SEM images of S45T45 hardened for 28 days, (**c**) shows the SEM images of S90T0 hardened samples at 90 days, (**d**–**h**) shows the SEM images of S45T45 hardened samples at 90 days, (**i**,**j**) shows the SEM and EDS of calcite phase in S45T45 hardened samples at 90 days, (**k**,**l**) shows the SEM and EDS of symbionts of C-S-H and calcite in S45T45 hardened samples at 90 days).

Figure 6 depicts the SEM images of S45T45 at different ages and S90T0 at 90 days. Figure 6a shows that in S45T45 at 7 d, particles are relatively loose and few hydration products cannot bond particles into an integral. Among loose particles, needle-like ettringite crystals are developed, which can strengthen hardened pastes to some extent.

Figure 6b shows that, in S45T45 at 28 d, the particles react to create a hardened paste base and sheet-like hydrocalumite crystals are developed. Hydrocalumite is the primary phase of the chemical curing of chlorine ions. Compared to S45T45 at 7 d, the S45T45 at 28 d has a more compact structure. This outcome reveals that, with the increase of curing age, the hydration degree increases continuously and the number of hydration products continues to increase. Binding materials produced by hydration bond solid particles into an integral, thus improving the strength of the hardened paste significantly.

Figure 6c shows that, in S90T0 at 90 days, the hardened body has a loose structure with more holes and poor integrity, while the S45T45 sample (Figure 6d) at the same age has a dense structure and significantly enhanced integrity.

Figure 6e–h exhibit that hydration is thorough in S45T45 at 90 d, and an integral with uniform texture has been formed. The structure tends to become more compact and the pilo-taxitic texture of fibrous-like (the sections of lamellar crystals) particles appears in the base. Figure 6e–g show that sheet-like hydration products buckle up into block-like pilotaxitic textures. These pilotaxitic textures are key to improving micro-texture uniformity and the strength of hardened pastes.

The component analysis of the block-like pilo-taxitic texture microcell in Figure 6i was conducted using EDS. The results showed that these block-like pilo-taxitic textures mainly comprise Ca, C and O (Figure 6j). Figure 6l shows CaO, C, Si and O are primary components of the thick lamellar crystal microcell in Figure 6k. The combination of XRD and the FTIR spectra of hardened pastes reveal that the block-like crystals in Figure 6i contain calcite. The thick lamellar crystal structure in Figure 6k shows the symbionts of C-S-H and calcite.

## 4. Conclusions

(1) Following the inclusion of TETS, the strength of hardened pastes is apparently improved, especially the early strength. The strength is enhanced with higher TETS content. The strength of S45T45 and S22.5T67.5 is increased to be equivalent to that of stable cement materials. They can serve as a sub-base for all roads. It is difficult for SS hardened pastes without TETS to meet the service requirements of the base and sub-base of roads.
(2) After adding TETS, the diffraction peaks of calcite and C-S-H in the hardened paste are strengthened. Moreover, there is a diffraction peak of hydrocalumite. The strength-increasing mechanism is primarily attributed to the combined effects of C-S-H gelation, calcite skeleton effect and hydrocalumite filling effect.
(3) When the SS/TETS ratio is equal to or greater than 45:45, prominent Portlandite diffraction peaks appear in hardened pastes. After TETS replaces some SS, the strength of hardened pastes increases without changing the type and content of activators, thus enabling them to meet the strength requirements of pavement base and sub-base for structural materials. In addition to lowering material costs, it consumes abundant SS and TETS. Hardened pastes with different SS/TETS ratios have low carbon, environmentally friendly, energy-saving and economic characteristics.

## Figures and Tables

**Table 1:** Chemical composition of raw materials used (%).

Composition	CaO	SiO_2_	Al_2_O_3_	TiO_2_	MgO	Cl	Fe_2_O_3_	SO_3_	F	MnO	K_2_O	Other
TETS	32.83	24.69	14.14	8.17	6.44	6.39	3.67	1.3	0.97	0.66	0.27	0.47
SS	45.36	16.08	5.07	1.34	8.93	0.38	15.94	0.89	-	2.83	0.35	2.87

**Table 2:** The ratio of raw materials for different hardened pastes.

Samples	CaO/%	SS/%	TETS/%
YS90T0	10.0	90.0	0.0
YS67.5T22.5	10.0	67.5	22.5
YS45T45	10.0	45.0	45.0
YS22.5T67.5	10.0	22.5	67.5
YS0T90	10.0	0.0	90.0

**Table 3:** MCSS for cement stabilized materials at 7 days and the ratio conforming to the standard.

No	Highway Grade and Structural Layer	Very Heavy, Heavy Traffic		Heavy Traffic		Medium and Light Traffic	
		MCSS (MPa)	The Ratio Conforming to the Standard	MCSS (MPa)	Meet the Required Ratio	MCSS (MPa)	The Ratio Conforming to the Standard
1	Freeways and primary highways base	≥5.0	S0T90	≥4.0	S0T90, S22.5T67.5	≥3.0	S0T90, S22.5T67.5, S45T45
2	Other grade highways base	≥4.0	S0T90, S22.5T67.5	≥3.0	S0T90, S22.5T67.5, S45T45	≥2.0	S0T90, S22.5T67.5, S45T45
3	Subbase of Freeways and primary highways	≥3.0	S0T90, S22.5T67.5, S45T45	≥2.5	S0T90, S22.5T67.5, S45T455	≥2.0	S0T90, S22.5T67.5, S45T45
4	Other grade highways subbase	≥2.5	S0T90, S22.5T67.5, S45T45	≥2.0	S0T90, S22.5T67.5, S45T45	≥1.0	S0T90, S22.5T67.5, S45T45, S67.5T22.5

**Table 4:** Summary of absorption peaks.

Absorption Peak	Absorption Peak Wave Numbers (cm^−1^)				
	1638–1634	1453–1400	1048–965	875	679–670
Mode of vibration	Bending vibration of H_2_O [40,41]	CO_3_^2−^ Stretching vibration of CO_3_^2−^ [42,43]	Stretching vibration of Si-O bond [19,20]	In-plane bending vibration of CO_3_^2−^ [40,44]	Symmetric stretching vibration of Si-O tetrahedron or T-O-T structure (T = Si, Al, Fe, Ti) [45]

## Data Availability

The authors confirm that the data supporting the findings of this study are available within the article.
